# Assessment of Fatty Acid Content in the Milk of Women from the West Pomeranian Region of Poland with Regard to Supplementation and the Amount of Adipose Tissue

**DOI:** 10.3390/nu15051110

**Published:** 2023-02-23

**Authors:** Dorota Ćwiek, Małgorzata Zimny, Katarzyna Szymoniak, Krystyna Czechowska, Olimpia Sipak-Szmigiel

**Affiliations:** Department of Obstetrics and Pathology of Pregnancy, Pomeranian Medical University in Szczecin, ul. Żołnierska 48, 71-210 Szczecin, Poland

**Keywords:** fatty acids (FA), breast milk, docosahexaenoic acid (DHA), body weight composition

## Abstract

The total amount of fat in the milk of nursing mothers depends on maternal reserves, as well as food intake and its synthesis in the mammary glands. The aim of this study was to assess the contents of fatty acids in the milk of women from the West Pomeranian region of Poland with regards to supplementation and the amount of adipose tissue. We also wanted to find out whether these women, who have direct access to the sea and potential access to fresh marine fish, have higher levels of DHA. Methods: We analyzed milk samples obtained 6–7 weeks postpartum from 60 women. The content of fatty acid methyl esters (FAME) in lipids was determined by gas chromatography–mass spectrometry (GC/MS) using a Clarus 600 device (PerkinElmer). Results: Women using dietary supplements had significantly higher levels of docosahexaenoic acid (DHA) (C22:6 n-3) (*p* = 0.000) and eicosapentaenoic acid (EPA) (20:5 n-3) (*p* = 0.000). The levels of eicosatrienoic acid (ETA) (C20:3 n-3) and γ-linolenic acid (GLA) increased with the amount of body fat, and the level of DHA was lowest in subjects where body fat exceeded 40% (*p* = 0.036). Conclusions: The contents of fatty acids in the milk of women from the West Pomeranian region of Poland were similar to that reported by other authors. The levels of DHA in women using dietary supplements was also comparable to the values reported worldwide. BMI had an effect on the levels of ETE and GLA acids.

## 1. Introduction

Breast milk is a species-specific substance equipped with all the nutrients and minerals, energy, hormones, and enzymes necessary for the baby’s body, as well as immune modulating, stimulating, and protective mechanisms. Feeding a child with breast milk is associated with an improved serum lipid profile, lower body weight, lower blood pressure, and a lower risk of developing insulin resistance later in life [1]. The contents of fatty acids in breast milk and the ratio of saturated fatty acids (SFAs) to monounsaturated fatty acids (MUFAs) are relatively constant. There are differences in the levels of long-chain polyunsaturated fatty acids (LC-PUFAs)—especially docosahexaenoic acid (DHA, C22:6 n-3) and arachidonic acid (ARA, C20:4 n-6)—which are extremely important for a rapidly developing baby [2]. LC-PUFAs are the main component of cell membrane phospholipids, neurons, and retinal receptors, and provide the cell membrane with optimal fluidity and integrity. By affecting neuronal membrane properties, they regulate the activity and function of membrane receptors and nerve conduction [3].

ARA and DHA regulate the expression of genes that are responsible for neuronal growth and interactions between nerve and glial cells. LC-PUFAs account for about 20% of dry weight of the brain, and the most intense accumulation of these acids in the human brain occurs in the postnatal period within 6–10 months after birth [4,5].

The total amount of fat in the milk of lactating mothers depends on maternal reserves, as well as food intake and its synthesis in the liver and mammary glands [6,7,8]. Adipose tissue in the hip and thigh area not only serves as an energy reserve for lactation but also as a reservoir of LC-PUFAs [9]. Nevertheless, enriching the diet of a nursing mother with fatty acids that are precursors of LC-PUFAs, i.e., linoleic acid (LA, C18:2 n-6) and α-linolenic acid (ALA, C18:3 n-3), allows the increase of LC-PUFA levels [10].

The aim of this study was to analyze the proportion of fatty acid content in the milk of women from the West Pomeranian Voivodeship and to assess the impact of supplementation and the amount of adipose tissue in women on these proportions.

## 2. Materials and Methods

### 2.1. Subjects and Study Design

One-hundred and six women in their second and third trimesters of pregnancy were invited to participate in the study. All women were recruited at the Outpatient Clinic for Pregnant Women and the Specialist State Clinical Hospital 1 in Police, Poland. The women were interviewed on sociodemographic data (age, education, place of residence, material status, employment status), as well as parity and prepregnancy body weight to assess prepregnancy body mass index (BMI). The women’s heights were also measured using a certified medical scale with a height measuring rod.

**Figure 1:**
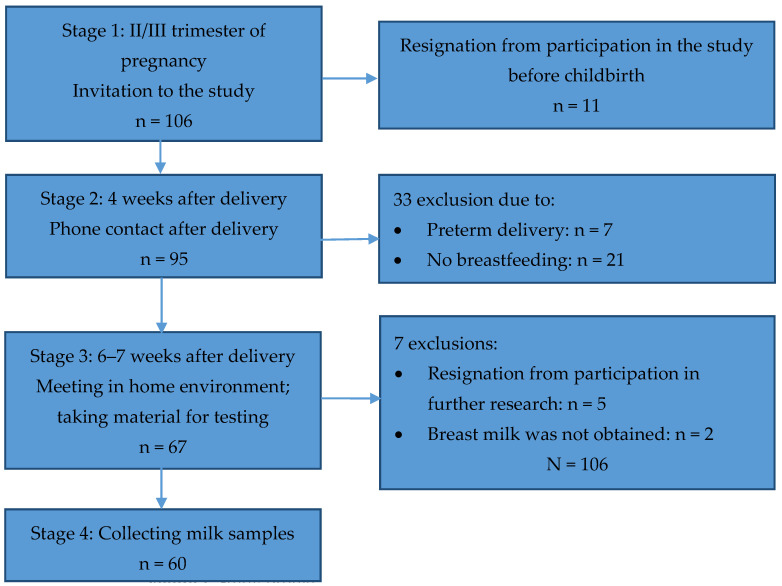
Study profile.

The actual examination was carried out 6–7 weeks after delivery. The inclusion criteria for the study were: age over 18 years, singleton pregnancy, giving birth to a child after the 37th week of pregnancy, and current breastfeeding. The exclusion criterion was mixed feeding in which formula was given at least four times a day or more. These criteria were met by 60 women. We used covariate adaptive randomization for this study (Figure 1).

Approximately 6–7 weeks after delivery, while visiting the patients’ homes, the following results were analyzed from the birth records: the time of delivery, the mothers’ weights before delivery, and the babies’ sex and birth weights. The women had their weight measured and body composition analyzed by means of the EU-certified (CE0122) TANITA DC-430 S MA body composition analyzer. The device met the Non-Automatic Weighing Instruments (NAWI) Directive and Class III standards for scales used for medical measurements, as well as the requirements of the Medical Device Directive (MDD 93/42/EEC). The women also had their heights measured in order to calculate their BMIs, which were then classified as proposed by the World Health Organization (WHO) [11].

### 2.2. Breast Milk Collection

To determine fat fractions in breast milk, 5 mL samples of the second-phase milk were collected from the women between 6:00 and 9:00 a.m. using the MEDELA Symphony breast pump, which were then secured in sealed polypropylene tubes, labeled, and delivered to the laboratory. The milk samples were immediately frozen at −20 °C then stored at −70 °C until further analysis in the laboratory of the Department of Poultry and Ornamental Bird Breeding of the West Pomeranian University of Technology in Szczecin.

### 2.3. Analysis of Lipid and Fatty Acid Levels in Breast Milk

Prior to analysis, the samples were thawed at room temperature in the absence of light and vortexed continuously for 5 min to ensure their homogeneity.

Extraction: The 500 µL milk samples were placed in 7.5 mL screw-cap orange glass vials with Teflon seals. A 5 mL mixture of chloroform and methanol with a volume ratio of 2:1 (Folch’s mixture) was added to each vial, nitrogen 5.0 was introduced, and the vials were sealed under a stream of nitrogen gas, followed by vigorous shaking for 3 h. To separate the chloroform phase from the non-lipid residue, the vials were centrifuged for 20 min at 2000 rpm. The same extraction step was repeated twice and the extracts were combined and filled to a final volume of 20 mL.

Hydrolysis: The chloroform phase equivalent to about 2 mg of extracted lipids was drawn into 4 mL amber glass vials. The vials were then sealed with Mininert valves (Supelco), allowing derivatization reagents to be dispensed under an inert gas atmosphere without the need to open the vials. Chloroform was evaporated from the extracts in a stream of nitrogen, then 400 µL of 0.5 M KOH solution in methanol was added to the dry residue and heated for 20 min in a heating block at 80 °C.

Esterification: After cooling, the vials were filled with 500 µL/l of a 14% solution of boron trifluoride (BF3) in methanol and incubated at 80 °C for 35 min. For more efficient extraction of fatty acid methyl esters (FAME), 1 mL of saturated NaCl solution and 2 mL of isooctane extractant were added to the cooled vials, shaken vigorously for 1 h, and left for 0.5 h until the phases separated. The upper isooctane layers were collected into separate vials, each containing about 0.5 g of anhydrous sodium sulfate (Na_2_SO_4_), and after filling the vials with nitrogen, they were left for 2 h. The dried FAME extracts were placed in vials into a gas chromatograph automatic sampler.

GC analysis: The FAME content in lipids was determined by gas chromatography–mass spectrometry (GC/MS) using a Clarus 600 device (PerkinElmer, Waltham, MA, USA) and the TC-80–60 m–0.25 mm–0.25 μm column (GL Sciences Inc., Tokyo, Japan). Standards of oleic acid, LA, ALA, GLA, conjugated linoleic acid (CLA) and a mixture of 37 fatty acids (Supelco TM 37 Component FAME Mix C4-C24) were used for analysis. Three repetitions were performed for all measurements.

Parameters of the GC method: carrier gas: helium (He) 6.0; gas flow through the column: 1 mL/min; volume: 1 μL; sample split in the dispenser (Split) 50:1; dispenser temperature: 200 °C; column temperature program: 110 °C held constant for 5 min, a linear gradient of 5 °C/min up to 180 °C; 180 °C constant for 15 min, a gradient of 5 °C/min up to 290 °C; 290 °C constant for 5 min; transfer line temperature: 290 °C.

Parameters of the MS method: Selected ion recording (SIR) analysis by selected ions (*m*/*z* abundance, (m—the mass of the ion; z—the charge of the ion)) for individual acids; ionization energy: 70 eV; ion source temperature: 200 °C; automatic optimization of MS ion optics.

The methodology was optimized based on the following standards:

- Vegetable and animal oils and fats. Sampling―PN-EN ISO 5555.
- Vegetable and animal oils and fats. Preparation of FAMEs―PN-EN ISO 5509.
- Analysis of FAMEs by gas chromatography―PN-EN ISO 5508.

The obtained results for the fatty acid content of breast milk are expressed as a percentage of weight by weight (% *w*/*w*) of fatty acids with a chain length of 8 to 24 carbon atoms (g/100 g of total FA).

The research protocol was approved by the Bioethics Committee of the Pomeranian Medical University in Szczecin (KB-0012/75/2015 of 22 June 2015 and KB-0012/61/2018 of 23 April 2018).

### 2.4. Statistical Analysis

Statistical analysis was performed using the licensed Statistica 13.0 software (StatSoft, Inc., Tulsa, OK, USA). The Shapiro–Wilk test was used to check the normality of the distribution of the examined variables. Homogeneity of variance was assessed by Levene’s test. Descriptive statistics were used to present the characteristics of the group, mainly means, standard deviations, and medians, as well as cardinality and percentages. The Mann–Whitney U test was used to analyze quantitative data in two groups, and the Kruskal–Wallis test in three groups. The level of significance was set as *p* ≤ 0.05.

## 3. Results

### 3.1. Maternal Characteristics

The study involved 60 women. Their mean chronological age was 31.52 years (min 19 years, max 45 years, standard deviation 4.76). Most of the women had third-level education (85.0%; n = 51), were married (71.7%; n = 43), and had a good material status (65.0%; n = 39). Most of the respondents lived in the city (91.7%; n = 55) and were employed (81.7%; n = 49). More than half of the women (58.3%; n = 35) reported having only one child, and nearly half had had a C-section (48.3%; n = 29). Of the 60 women, 68.3% were healthy and 31.7% had had chronic diseases treated before pregnancy. The most common health problems were hypothyroidism (18.3%), asthma (1.7%), epilepsy (1.7%), thromboembolism (1.7%), anemia (1.7%), thrombophilia (1.7%), Crohn’s disease (1.7%), Sjögren’s syndrome (1.7%), and gastric hernia (1.7%) (Table 1).

The mean body weight of the women before pregnancy was *M* = 67.28 kg, before delivery was *M* = 79.45 kg, and 6–8 weeks after delivery was *M* = 69.16 kg. The subjects’ BMI calculated 6–7 weeks after delivery was *M* = 25.33. The mean percentage of fat in the body was 32.51%, and the percentage of water was 47.58%. The mean waist circumference was *M* = 89.07 cm, and the mean degree of obesity was 15.14% (Table 2).

### 3.2. Fatty Acid Profile

Analysis of the percentages of fatty acid content of breast milk revealed that MUFAs constituted 42.36%, SFAs—39.783%, and PUFA—17.64%. Palmitic (C16:0–17.67%) and stearic (C18:0–6.6%) acids were the most abundant SFAs, and oleic acid (C18:1 n-9c—38.0%) was the most abundant MUFA. The total level of n-3 acids was 2.91%, with ALA (C18:3 n-3—1.37%) being the most prevalent. DHA (C22: 6 n-3) was present in the amount of 0.66%, and eicosapentaenoic acid (EPA, C20:5 n-3)—0.101%. The total amount of n-6 acids was 14.73%, of which LA (C18:2 n-6c) accounted for 13.68% and ARA (C20:4 n-6) for 0.668%. The ratio of n-6 to n-3 acids was 5.469 (Table 3 and Table 4).

Table 5 shows the levels of fatty acids in breast milk depending on postpartum supplementation with DHA or DHA + EPA. Women taking dietary supplements had significantly higher levels of DHA (0.76% vs. 0.29%) and EPA (0.12% and 0.03%). It was also noted that women not using supplementation had significantly higher levels of oleic acid (40.7% vs. 37.31%; *p* = 0.012).

We analyzed body weight composition, including the percentage of body fat. The level of eicosatrienoic acid (ETA) (C20: 3 n3) from the n-3 group increased with the amount of adipose tissue. The lowest levels were found in women with up to 29.9% body fat, and the highest levels in those with more than 40% body fat (0.76% and 0.93%, respectively; *p* = 0.048). The level of DHA was highest in women with 30.0–39.9% body fat, and lowest in those with over 40% body fat (0.79 vs. 0.43; *p* = 0.036). The level of γ-linolenic acid (GLA) increased with the amount of body fat: the lowest levels were observed in women with up to 34.9% of adipose tissue and the highest in those with more than 40.0% of body fat (0.04 vs. 0.06; *p* = 0.033). No significant differences were noted for SFAs or MUFAs. Women with the lowest percentage of body fat (up to 29.9%) had a significantly higher ratio of n-6 to n-3 than those with more body fat (35.0–39.9%) (6.27 vs. 4.76; *p* = 0.034) (Table 6).

The group with BMI up to 18.5 included only one woman, and hence it was not representative and was not taken into account. There were no statistically significant differences in the levels of SFAs or MUFAs. Significant differences were only observed in ETA and GLA fractions. The levels of ETA (C20: 3 n-3) increased with BMI values. The lowest level was found in women with normal body weight (BMI 18.6–25.0), and the highest in those with obesity (BMI > 30.0) (0.72% and 0.9%, respectively; *p* = 0.011). Levels of GLA increased with the values of BMI, the lowest being in women with normal body weight (BMI 18.6–25.0) and the highest in obese women (BMI > 30.0) (0.04 and 0.06, respectively; *p* = 0.021). The ratio of n-6 to n-3 was significantly higher in women with normal body weight than in overweight and obese ones (6.12 vs. 4.67 and 4.87, respectively; *p* = 0.020) (Table 7).

An increase in BMI was accompanied by a linear increase in the amount of C20:3 n-3 (11(Z),14(Z),17(Z)-eicosatrienoic acid) andC18:3 n-6 (GLA/6(Z),9(Z),12(Z)-octadecatrienoic acid). As there was only one woman with BMI > 18.5, she was not included in the calculations.

Table 8 shows the content of fatty acids in relation to the presence of chronic diseases in the subjects. No statistically significant differences were observed (*p* < 0.05).

## 4. Discussion

The levels of fatty acids in breast milk depend on maternal reserves, diet, supplementation, and fatty acid synthesis in the mammary glands [6,12,13,14]. The mean total level of SFAs in our study was 39.78%, MUFAs—42.36%, and PUFAs—17.64% (including 14.73% of n-6 and 2.91% of n-3). These values are comparable to those obtained by Pedersen et al. In their study, SFAs accounted for 41.8%, MUFAs for 40.9%, and PUFAs for 13.7%. The n-6/n-3 ratio in our study was 5.07, while the one reported by Persden et al. was 5.7 [15]. Similar results were described by Sánchez-Hernández. In their study, total SFAs in breast milk was 33.19%, MUFAs was 46.99%, and PUFAs was 17.48% [16].

Considering the composition of breast milk, the highest percentages of SFAs were noted for palmitic (16:0; 17.67%) and stearic (C18:0; 6.6%) acids. The most abundant MUFA was oleic acid (C18:1 n-9c; 38.0%). Similar results were obtained 30 days after delivery by Giuffrida et al., who found that palmitic acid (C16:0) was the most abundant of all SFAs (22.39%), followed by stearic acid (C18:0; 6.39%). Oleic acid (18:1 n-9) was the most abundant of all MUFAs (35.72%) [17]. Slightly different results were obtained by Samur et al., who reported that palmitic, stearic, and oleic acids accounted for 20.9%, 5.66% and 27.31%, respectively [18].

The greatest interest among researchers, however, has been aroused by the contents of unsaturated acids (PUFAs) in breast milk. The synthesis of DHA in the mammary gland is minimal, so its concentration depends mainly on a woman’s eating habits, including the consumption of fish and dietary supplements. Marine mammals contain large amounts of DHA and are therefore a rich source of this fatty acid [19]. Dietary intake of DHA varies in many parts of the world. According to Ogunleye et al., the lowest DHA levels in breast milk (0.06–0.14%) were observed in landlocked and developed countries, which was usually associated with low consumption of seafood [20]. Our study showed that breast milk contained 2.11% of all n-3 fatty acids, with ALA (C18:3 n-3; 1.37%) being the most abundant, followed by ETA (C20:3 n-3; 0.78%), DHA (C22:6 n-3; 0.66%), and EPA (C20:5 n-3; 0.101%). Among n-6 fatty acids, the highest levels were recorded for LA (C18:2 n-6c; 13.68%) and ARA (C20:4 n-6; 0.668%). All n-6 acids accounted for 14.73%, and the ratio of n-6 to n-3 was 5.07. In a study by Giuffrida et al., the most abundant n-6 PUFA (14.94% of all FAs) one month after delivery was LA (13.53%), and in the n-3 group (1.39% of all FAs) ALA (0.87%) and DHA (0.6%) [17]. Siziby et al. observed higher levels of n-6 in breast milk (22.3%), but lower levels of n-3 (1.4%) and a significantly higher ratio of omega 6 to omega 3 (15.4) [21]. In a cross-country review by Sauerwald et al., LA ranged from 6.9% (United Kingdom) to 20.6% (China), ARA from 0.4% (Germany) to 0.9% (China); ALA from 0.4% (South Africa) to 3.0% (China) and DHA from 0.1% (Hungary) to 0.9% (China) [22]. In a comprehensive review of peer-reviewed publications by Fu et al., the mean levels of DHA and ARA worldwide were 0.32% and 0.47%, respectively [23], while the mean levels of DHA and ARA in breast milk in a study by Jimenez et al. were 0.73% and 0.69%, respectively [24].

Brenna et al. conducted a systematic review of studies on the levels of DHA and ARA in breast milk worldwide, published between 1986 and 2006. The lowest DHA levels were 0.06% (Pakistan), and the highest were 1.4% (Canadian Arctic) and 1.1% (Japan). The authors concluded that fatty acid concentrations in breast milk varied depending on the region of residence and the lifestyle of the nursing mothers. They also found that the average concentration of DHA in breast milk was 0.32 ± 0.22% of all fatty acids. In comparison, in our study, the concentration of DHA in the milk of women taking dietary supplements averaged 0.76% of all fatty acids which was twice as high as the average value from the review studies [25]. Poland was not included in this compilation. We did not have data on women from northwestern Poland, with easy access to seafood. However, we found one similar study conducted in central Poland. Its authors concluded that food sources of omega-3 fatty acids (mainly fish) are not consumed on a daily basis and are not responsible for their content in breast milk. In our study, we did not assess the effect of diet on fatty acid levels, but the West Pomeranian Voivodeship is a coastal region with easy access to fresh sea fish. It can therefore be expected that DHA levels should be higher, as shown in many studies of coastal areas with easy access to marine products [20,23,24,25]. However, DHA in breast milk of women from central Poland constituted 0.7 ± 0.3% of total fatty acids, so it was a very similar result to ours [26]. A study by Kim et al. showed that the mean n-3 content in breast milk was 3.0% and n-6 content was 18.2%, with ARA, EPA and DHA containing 0.48%, 0.15% and 0.67% of the total fatty acids, respectively [27].

Wang et al. found that Japanese and Chinese women had similar ARA levels (1.0% and 0.8%, respectively) [28]. In our study, the level of ARA was slightly lower (0.64%). According to Kim et al., the levels of n-3 were significantly higher in breastfeeding women who used supplements than in those who did not [27]. In addition, in the groups receiving supplementation, Helland et al. states that there was a significant increase in DHA and EPA levels and overall increased total level of n-3 and n-6 [29]. This is also confirmed by our research: the values of DHA in mothers who supplemented this compound were significantly higher than in those without supplementation (0.76% vs. 0.29%; *p* <0.001). Similar results were achieved for EPA: 0.76% vs. 0.29% (*p* < 0.001).

Bortolozo et al. studied the impact of omega-3 fatty acid supplementation in the third trimester of pregnancy and after childbirth on breast milk composition. There was no statistical difference in total lipid values between the study groups, but the milk of mothers supplemented with fish oil had higher concentrations of DHA and EPA. Thus, it was concluded that a higher intake of omega-3 fatty acids affects their concentration in breast milk [13]. In addition, Puca et al. analyzed seven articles from 2010–2020 on supplementation during pregnancy and lactation. Based on these studies, they concluded that supplementation with omega-3 fatty acids in the form of DHA or DHA + EPA in appropriate proportions has a positive effect on the content of fatty acids in breast milk without reducing the levels of ARAs [30]. In a systematic review by Notarbartolo et al., all studies demonstrated a positive association between eating foods rich in omega-3 fatty acids and their concentrations in breast milk, hence emphasizing the importance and safety of supplementation of these fatty acids during pregnancy and/or puerperium [31]. Similar conclusions were reached by Much et al., who found that dietary intervention resulted in significantly higher levels of DHA and EPA in women than those who did not receive supplementation (1.34% vs. 0.28% for DHA and 0.18% vs. 0.08% for EPA) [32]. A study by Mäkelä et al. showed that women consuming dietary supplements had higher PUFA levels (15.2% vs. 13.7%, *p* = 0.044), especially n-3 (2.8% vs. 2.3%, *p* = 0.030) in breast milk than women not consuming FA supplements [33]. In addition, Sherry et al. states that mothers supplemented with DHA have over double the DHA levels of those without supplementation. This was not observed for the entire n-3 group, but n-6 was significantly higher in women without supplementation [19]. As stated by Warstedt et al., the levels of DHA and the entire n-3 fraction in breast milk of supplementing women were significantly higher than in their non-supplementing counterparts (1.1% vs. 0.27%) [34]. Poland is one of the countries with low fish consumption. Therefore, the levels of n-3 in women not using supplements are quite low compared to those reported by other authors. According to Wang et al., the level of DHA in the milk of Japanese women was twice as high as in Chinese women and seven times as high as in Canadian women. Polish women who did not use dietary supplements had DHA levels comparable to Canadian women (0.29%) [28]. Fu et al. believes that residents of high-income countries tend to eat more processed foods and trans fats, which may affect the synthesis and metabolism of long-chain fatty acids [23]. This is confirmed by a study by Xiang et al., in which SFAs and MUFAs in the milk of Chinese mothers were significantly lower than in the milk of Swedish mothers. In the milk of Chinese mothers, the levels of all n-6 PUFAs, except for GLA (C18:3 w-6) and docosapentaenoic acid (DPA, C22:5 w-6), were significantly higher than in the milk of Swedish mothers. However, the levels of all n-3 PUFAs, except for ETA (C20:3 n-3), were significantly lower than in the milk of Swedish women [35].

Many researchers have tried to find out whether body weight composition or the weight of lactating women affects the contents of fatty acids. Mäkelä et al. observed that the milk of overweight women had significantly more SFAs (*p* = 0.012) and less n-3 acids (*p* = 0.010) and that the ratio of unsaturated to saturated fatty acids was lower and the ratio of n-6 to n-3 higher than in normal-weight women [33]. In addition, a study by Storck Lindholm et al. revealed significantly higher DHA levels in normal-weight women than in obese ones. Unfortunately, our study did not show significant differences in DHA between the groups of women with differing BMI. However, obese women had the lowest concentrations of DHA in breast milk [36]. Similar conclusions were reached by Kim et al., who found that the contents of individual groups of fatty acids (SFA, MUFA, PUFA) in breast milk were not correlated with maternal BMI [27]. In a study by Miliku et al., maternal body weight was related to the contents of fatty acids in breast milk. They found that compared to normal-weight mothers, overweight and obese mothers had +0.32 SD and +0.39 SD higher total SFA, respectively. Being overweight or obese in their study was also negatively associated with a total MUFA to ARA ratio. The amount of DHA was lower in obese mothers—a finding also supported by our study—although the difference was not statistically significant [37]. Marın et al. noted that the total lipid content of milk samples from obese mothers showed significantly higher levels than the normal- and overweight groups. The total level of MUFAs was significantly lower in overweight and obese mothers than in those with normal weight. There was also a significant increase in the total content of PUFAs in the milk of obese mothers compared to women with normal body weight. In contrast, there were no differences in the proportion of n-3 fatty acids [38]. In addition, in the study by Nayak et al. there was no relationship between maternal BMI and SFA/PUFA ratios or n-6/n-3 ratios [39].

In addition, we analyzed the amount of CLA, which is the most common conjugated LA isomer from the group of unsaturated fatty acids, found mainly in dairy products and beef. It has been shown to be beneficial for health due to its strong antioxidant effect (inhibition of cancer cell proliferation), and its effect on body composition and fat metabolism (inhibition of lipogenesis, and thus the lowering of the content of triglycerides, total cholesterol, and LDL fractions in the blood). Consequently, it has antiatherosclerotic, antidiabetic, and immunomodulatory properties [40]. Nishimura’s research showed that the mean value of CLA was 0.02 [41], which was similar to that in our study (0.165). The main sources of these fatty acids are dairy products, but as noted by Precht et al., the contents of these fatty acids may vary depending on how cattle are raised [42]. In another Polish study, the mean content of total CLA in the milk of women following a diet without limiting the consumption of milk and milk products was 0.4% (range from 0.32 to 0.57), while those on a diet poor in milk and milk products was 0.22% (range from 0.14 to 0.33) [43]. In our study, a different result was obtained—0.165 (range from 0.019 to 0.598).

## 5. Conclusions

The contents of fatty acids in milk of Polish women from the West Pomeranian region were similar to those reported by other authors. In addition, the level of DHA in women using dietary supplements was comparable to the reported global values. BMI had an impact on the levels of ETA and GLA. Further research is needed to determine to what extent a mother’s diet during pregnancy and after delivery affects the fatty acid contents of their milk.

Limitations of this study, such as the low sample size and its ethnic homogeneity, should be taken into account. Poland is one of the countries with low ethnic diversity.

## Figures and Tables

**Table 1:** Characteristics of the study sample.

Characteristics of the Study Sample		n = 60	
		n	%
Education	Vocational	0	0
	Primary	0	0
	Secondary	9	15.0
	Third-level	51	85.0
Marital status	Married	43	71.7
	Single	17	28.3
Place of residence	Village	5	8.3
	City	55	91.7
Number of children	1	35	58.3
	2	17	28.3
	3	7	11.7
	4	1	1.7
Financial standing	Bad	0	0
	Average	6	10.0
	Good	39	65.0
	Very good	15	25.0
Employment status	Employed	49	81.7
	Unemployed	10	16.7
	Student	1	1.7
Type of delivery	Vaginal birth	31	51.7
	C-section	29	48.3
Women’s health	Without chronic diseases treated before pregnancy	41	68.3
	With chronic diseases treated before pregnancy	19	31.7

n—number of respondents.

**Table 2:** Anthropometric data.

Anthropometric Data	Mean ± SD	Me (Min–Max)
Prepregnancy weight [kg]	67.28 ± 13.09	65.25 (45.0–100.0)
Prepartum weight [kg]	79.45 ± 13.42	77.00 (56.0–120.0)
Weight gain during pregnancy [kg]	12.00 ± 6.80	11.25 (−2.0–30.0)
Current body weight [kg]	69.16 ± 12.42	67.00 (50.0–107.8)
BMI	25.33 ± 4.35	24.55 (17.3–37.3)
Waist circumference [cm]	89.07 ± 11.72	87.75 (33.5–116.5)
% of fat	32.51 ± 6.74	32.45 (21.1–49.4)
Fat mass [kg]	23.26 ± 9.01	22.25 (10.7–53.3)
Lean body mass [kg]	45.93 ± 4.27	44.95 (39.3–58.4)
% of water (total body water, TBW)	47.58 ± 4.16	47.50 (37.3–54.6)
Degree of obesity [%]	15.14 ± 19.80	11.65 (−21.4–69.5)

SD—standard deviation, Min—minimum, Max—maximum, Me—median; BMI—body mass index.

**Table 3:** Fatty acid content of breast milk (g/100 g of total FA).

Fatty Acids	Mean ± SD	Me (Min–Max)
Saturated fatty acids (SFA)		
C8:0—caprylic acid	0.160 ± 0.070	0.153 (0.008–0.333)
C10:0—capric acid	1.83 ± 0.473	1.802 (0.743–2.961)
C12:0—lauric acid	5.54 ± 1.426	5.632 (2.280–8.923)
C14:0—myristic acid	6.19 ± 1.300	6.089 (3.709–9.120)
C15:0—pentadecanoic acid	0.43 ± 0.198	0.407 (0.078–1.001)
C16:0—palmitic acid	17.67 ± 2.426	17.381 (12.060–27.607)
C17:0—margaric acid	0.84 ± 0.192	0.827 (0.401–1.363)
C18:0—stearic acid	6.60 ± 1.024	6.472 (4.029–9.402)
C20:0—arachidic acid	0.131 ± 0.072	0.114 (0.008–0.403)
C24:0—lignoceric acid	0.39 ± 0.093	0.393 (0.021–0.641)
Total saturated fatty acids (SFAs)	39.783 ± 5.060	39.254 (27.109–53.565)
Monounsaturated fatty acids (MUFAs)		
C14:1—myristoleic acid	0.31 ± 0.176	0.275 (0.044–0.743)
C16:1—palmitoleic acid	2.82 ± 0.596	2.819 (1.472–4.420)
C18:1 n-9c—oleic acid	38.0 ± 3.959	37.518 (30.512–49.667)
C18:1 n-9t—elaidic acid	0.22 ± 0.124	0.193 (0.037–0.806)
C20:1—eicosenoic acid	0.77 ± 0.231	0.730 (0.392–1.821)
C22:1 n-9—erucic acid	0.24 ± 0.076	0.231 (0.069–0.508)
Total monounsaturated fatty acids (MUFAs)	42.36 ± 3.981	42.283 (33.039–53.021)
n-3 polyunsaturated fatty acids (n-3 PUFAs)		
C18:3 n-3—(ALA) α-linolenic acid	1.37 ± 0.676	1.256 (0.354–3.649)
(ETA) C20:3 n-3—eicosatrienoic acid	0.78 ± 0.186	0.776 (0.358–1.249)
C20:5 n-3—(EPA) eicosapentaenoic acid	0.101 ± 0.130	0.054 (0.013–0.644)
C22:6 n-3—(DHA) docosahexaenoic acid	0.66 ± 0.408	0.541 (0.146–1.902)
Total n-3 polyunsaturated fatty acids (n-3 PUFAs)	2.911 ± 0.872	2.945 (1.459–5.427)
n-6 polyunsaturated fatty acids (n-6 PUFAs)		
C18:2 n-6c—(LA) linoleic acid	13.68 ± 2.391	13.458 (8.667–20.873)
C18:3 n-6—(GLA) γ-linolenic acid	0.0443 ± 0.028	0.038 (0.000–0.134)
C20:2—(all Z)-eicosadienoic	0.30 ± 0.088	0.281 (0.033–0.555)
C20:3 n-6—dihomo-γ-linoleic acid	0.04 ± 0.026	0.034 (0.001–0.124)
C20:4 n-6—(ARA) arachidonic acid	0.668 ± 0.153	0.670 (0.253–0.971)
Total n-6 polyunsaturated fatty acids (n-6 PUFAs)	14.7323 ± 2.488	14.521 (9.385–22.350)
Total polyunsaturated fatty acids	17.6433 ± 2.854	17.492 (10.844–26.152)
Other		
Conjugated linoleic acid (CLA) (Z,E)-9,11-octadecadienoic acid	0.165 ± 0.139	0.123 (0.019–0.598)
Other	0.165 ± 0.139	

SD—standard deviation, Min—minimum, Max—maximum, Me—median.

**Table 4:** Groups of fatty acids (g/100 g of total FA).

Groups of Fatty Acids	Mean ± SD	Me (Min–Max)
Saturated fatty acids (SFAs)	39.783 ± 5.060	39.254 (27.109–53.565)
Total monounsaturated fatty acids (MUFAs)	42.36 ± 3.981	42.283 (33.039–53.021)
Total polyunsaturated fatty acids (PUFAs)	17.6433 ± 2.854	17.492 (10.844–26.152)
- total n-3 polyunsaturated fatty acids (n-3 PUFAs)	2.911 ± 0.872	2.945 (1.459–5.427)
- total n-6 polyunsaturated fatty acids (n-6 PUFAs)	14.7323 ± 2.488	14.521 (9.385–22.350)
PUFA/SFA	0.456 ± 0.120	0.452 (0.214–0.877)
PUFA/MUFA	0.419 ± 0.082	0.405 (0.285–0.711)
MUFA/SFA	0.956 ± 0.203	0.936 (0.528–1.621)
n-6/n-3	5.469 ± 1.739	5.196 (2.996–11.294)

SD—standard deviation, Min—minimum, Max—maximum, Me—median.

**Table 5:** Fatty acid content of breast milk (g/100 g of total FA) depending on supplementation with DHA or DHA + EPA.

Fatty Acids	No Supplementation (n = 13)		Supplementation with DHA or DHA + EPA (n = 47)		*p*
	Mean ± SD	Me (Min–Max)	Mean ± SD	Me (Min–Max)	
SFA	38.03 ± 6.31	38.28 (27.97–50.61)	40.27 ± 4.62	39.89 (27.11–53.56)	0.123
C8:0—caprylic acid	0.15 ± 0.06	0.15 (0.66–0.239)	0.16 ± 0.07	0.15 (0.008–0.333)	0.914
C10:0—capric acid	1.75 ± 0.43	1.75 (1.06–2.50)	1.85 ± 0.48	1.85 (0.74–2.96)	0.379
C12:0—lauric acid	5.32 ± 1.70	5.18 (2.95–7.83)	5.60 ± 1.35	5.64 (2.28–8.92)	0.542
C14:0—myristic acid	5.68 ± 1.54	5.50 (3.74–8.87)	6.34 ± 1.20	6.32 (3.71–9.12)	0.106
C15:0—pentadecanoic acid	0.37 ± 0.21	0.34 (0.08–0.95)	0.45 ± 0.19	0.43 (0.15–1.0)	0.141
C16:0—palmitic acid	17.16 ± 2.91	16.74 (12.78–22.4)	17.81 ± 2.29	17.53 (12.06–27.61)	0.333
C17:0—margaric acid	0.78 ± 0.24	0.77 (0.4–1.36)	0.86 ± 0.17	0.89 (0.41–1.33)	0.103
C18:0—stearic acid	6.34 ± 1.22	6.31 (4.78–8.35)	6.67 ± 0.96	6.47 (4.03–9.4)	0.351
C20:0—arachidic acid	0.11 ± 0.05	0.11 (0.04–0.22)	0.14 ± 0.08	0.12 (0.01–0.4)	0.495
C24:0—lignoceric acid	0.36 ± 0.08	0.38 (0.21–0.49)	0.39 ± 0.10	0.39 (0.02–0.64)	0.324
MUFA	44.96 ± 4.22	46.22 (38.07–53.02)	41.70 ± 3.65	41.66 (33.04–48.93)	0.012 *
C14:1—myristoleic acid	0.27 ± 0.18	0.27 (0.04–0.74)	0.32 ± 0.18	0.29 (0.06–0.72)	0.333
C16:1—palmitoleic acid	2.85 ± 0.57	2.75 (2.13–4.29)	2.81 ± 0.61	2.84 (1.47–4.42)	0.957
C18:1 n-c—oleic acid	40.70 ± 4.64	41.67 (33.1–49.7)	37.31 ± 3.46	37.33 (30.5–44.9)	0.012 *
C18:1 n-9t—elaidic acid	0.19 ± 0.07	0.18 (0.08–0.35)	0.23 ± 0.13	0.20 (0.04–0.81)	0.615
C20:1—eicosenoic acid	0.73 ± 0.15	0.71 (0.5–1.0)	0.78 ± 0.25	0.73 (0.39–1.82)	0.615
C22:1 n-9—erucic acid	0.22 ± 0.05	0.21 (0.16–0.35)	0.25 ± 0.08	0.23 (0.07–0.51)	0.073
n-3	2.33 ± 0.83	2.14 (1.46–4.18)	3.08 ± 0.82	3.25 (1.76–5.43)	0.005 *
C18:3 n-3—(ALA) α- linolenic acid	1.25 ± 0.79	1.08 (0.35–3.17)	1.41 ± 0.65	1.30 (0.49–3.65)	0.251
C20:3 n-3—(ETA) eicosatrienoic acid	0.75 ± 0.11	0.76 (0.58–0.95)	0.79 ± 0.20	0.78 (0.36–1.25)	0.733
C20:5 n-3—(EPA) eicosapentaenoic acid	0.03 ± 0.02	0.03 (0.016–0.066)	0.12 ± 0.14	0.07 (0.013–0.644)	0.000 *
C22:6 n-3—(DHA) docosahexaenoic acid	0.29 ± 0.08	0.27 (0.15–0.40)	0.76 ± 0.40	0.70 (0.16–1.90)	0.000 *
n-6	14.55 ± 2.42	15.18 (9.39–17.63)	14.78 ± 2.53	14.41 (10.58–22.35)	0.720
C18:2 n-6c—(LA) linoleic acid	13.53 ± 2.29	13.86 (8.7–16.5)	13.72 ± 2.44	13.23 (9.8–20.9)	0.680
C18:3 n-6—(GLA) γ-linolenic acid	0.05 ± 0.03	0.05 (0.088–0.0151)	0.04 ± 0.03	0.03 (0.0000–0.1338)	0.542
C20:2—(all Z)-eicosadienoic	0.29 ± 0.08	0.27 (0.18–0.48)	0.30 ± 0.09	0.29 (0.03–0.55)	0.440
C20:3 n-6—dihomo-γ-linoleic acid	0.04 ± 0.02	0.03 (0.01–0.08)	0.04 ± 0.03	0.03 (0.00–0.12)	0.379
C20:4 n-6—(ARA) arachidonic acid	0.64 ± 0.15	0.67 (0.413–0.857)	0.67 ± 0.16	0.67 (0.253–0.971)	0.542
PUFA	16.88 ± 2.98	17.54 (1.46–17.63)	17.86 ± 2.82	17.45 (1.76–22.35)	0.654
Other					
Conjugated linoleic acid (CLA) (Z,E)-9,11-octadecadienoic	0.13 ± 0.13	0.08 (0.019–0.475)	0.17 ± 0.14	0.13 (0.027–0.598)	0.243
Proportions					
PUFA/SFA	0.46		0.45		0.872
PUFA/MUFA	0.38		0.43		0.025 *
MUFA/SFA	0.86		0.98		0.043 *
n-6/n-3	6.75		5.11		0.004 *

SD—standard deviation, Min—minimum, Max—maximum, Me—median, *—significant differences (*p* < 0.05).

**Table 6:** Fatty acid content of breast milk (g/100 g of total FA) depending on the amount of body fat.

Fatty Acids	% of Fat (<29.9%) n = 23		% of Fat (30.0–34.9%) n = 15		% of Fat (35.0–39.9%) n = 14		% of Fat (>40.0%) n = 8		*p*
	Mean ± SD	Me (Min–Max)	Mean ± SD	Me (Min–Max)	Mean ± SD	Me (Min–Max)	Mean ± SD	Me (Min–Max)	
SFA	40.51 ± 5.77	40.72 (27.97–53.56)	40.24 ± 4.24	39.33 (32.90–48.11)	38.05 ± 5.22	38.12 (27.11–47.81)	39.89 ± 4.04	39.52 (33.52–46.17)	0.498
C8:0—caprylic acid	0.16 ± 0.08	0.14 (0.065–0.307)	0.13 ± 0.06	0.14 (0.08–0.278)	0.18 ± 0.07	0.17 (0.77–0.333)	0.16 ± 0.05	0.16 (0.066–0.235)	0.280
C10:0—capric acid	1.90 ± 0.52	1.76 (0.95–2.96)	1.62 ± 0.41	1.73 (0.74–2.41)	1.93 ± 0.36	1.95 (1.41–2.63)	1.86 ± 0.56	1.88 (1.06–2.67)	0.304
C12:0—lauric acid	5.89 ± 1.42	6.12 (2.95–7.83)	5.04 ± 1.30	5.20 (2.28–7.57)	5.47 ± 1.44	4.89 (3.85–8.92)	5.58 ± 1.63	5.25 (3.38–7.80)	0.365
C14:0—myristic acid	6.42 ± 1.36	6.38 (3.74–8.87)	6.19 ± 1.08	6.32 (3.71–7.64)	5.80 ± 1.31	5.76 (3.91–8.53)	6.25 ± 1.58	5.71 (4.18–9.12)	0.501
C15:0—pentadecanoic acid	0.39 ± 0.21	0.35 (0.08–0.95)	0.52 ± 0.21	0.49 (0.18–1.0)	0.40 ± 0.18	0.35 (0.15–0.73)	0.42 ± 0.15	0.39 (0.27–0.71)	0.182
C16:0—palmitic acid	17.73 ± 3.06	17.19 (12.78–27.61)	18.43 ± 1.87	17.82 (16.25–22.40)	16.77 ± 2.07	16.89 (12.06–19.74)	17.64 ± 1.54	18.24 (15.33–19.33)	0.307
C17:0—margaric acid	0.83 ± 0.22	0.90 (0.40–1.36)	0.90 ± 0.22	0.90 (0.41–1.33)	0.82 ± 0.15	0.79 (0.58–1.03)	0.82 ± 0.12	0.77 (0.72–1.03)	0.631
C18:0—stearic acid	6.69 ± 1.30	6.47 (4.78–9.40)	6.91 ± 0.63	6.84 (6.11–8.27)	6.11 ± 0.91	6.35 (4.03–7.37)	6.60 ± 0.72	6.36 (5.64–7.62)	0.224
C20:0—arachidic acid	0.11 ± 0.06	0.10 (0.04–0.25)	0.13 ± 0.07	0.12 (0.01–0.26)	0.16 ± 0.09	0.15 (0.03–0.40)	0.14 ± 0.04	0.13 (0.09–0.20)	0.137
C24:0—lignoceric acid	0.39 ± 0.09	0.37 (0.21–0.64)	0.35 ± 0.11	0.38 (0.02–0.49)	0.41 ± 0.09	0.41 (0.28–0.64)	0.42 ± 0.06	0.41 (0.34–0.56)	0.614
MUFA	41.37 ± 4.80	41.48 (33.04–53.02)	42.31 ± 3.23	41.81 (36.80–48.26)	43.67 ± 3.29	43.44 (37.99–48.93)	43.35 ± 3.50	42.48 (39.28–48.32)	0.315
C14:1—myristoleic acid	0.27 ± 0.19	0.19 (0.04–0.74)	0.38 ± 0.18	0.38 (0.08–0.72)	0.29 ± 0.17	0.26 (0.06–0.61)	0.34 ± 0.14	0.30 (0.18–0.63)	0.110
C16:1—palmitoleic acid	2.68 ± 0.59	2.60 (1.47–3.86)	2.96 ± 0.58	2.89 (1.88–4.29)	2.82 ± 0.75	2.50 (1.99–4.42)	2.96 ± 0.24	3.01 (2.57–3.33)	0.402
C18:1 n-9c—oleic acid	37.18 ± 4.70	36.23 (30.5–49.7)	37.74 ± 3.31	37.39 (31.9–43.1)	39.28 ± 3.38	39.46 (32.4–44.9)	38.90 ± 3.59	38.16 (34.5–44.2)	0.329
C18:1 n-9t—elaidic acid	0.19 ± 0.10	0.16 (0.06–0.51)	0.27 ± 0.19	0.20 (0.04–0.81)	0.21 ± 0.05	0.22 (0.12–0.28)	0.24 ± 0.10	0.21 (0.12–0.45)	0.309
C20:1—eicosenoic acid	0.79 ± 0.30	0.72 (0.39–1.82)	0.72 ± 0.25	0.65 (0.47–1.45)	0.83 ± 0.11	0.81 (0.70–1.0)	0.72 ± 0.09	0.72 (0.61–0.83)	0.140
C22:1 n-9—erucic acid	0.26 ± 0.09	0.23 (0.6–0.51)	0.24 ± 0.08	0.23 (0.07–0.47)	0.24 ± 0.06	0.24 (0.16–0.42)	0.20 ± 0.02	0.20 (0.17–0.23)	0.198
n-3	2.63 ± 0.83	2.82 (1.46–4.18)	3.01 ± 0.76	3.25 (1.64–4.74)	3.35 ± 0.99	3.30 (1.89–5.43)	2.79 ± 0.75	2.74 (1.62–4.14)	0.179
C18:3 n-3—(ALA) α- linolenic acid	1.20 ± 0.64	0.97 (0.35–3.17)	1.40 ± 0.65	1.31 (0.49–2.48)	1.64 ± 0.84	1.58 (0.51–3.65)	1.35 ± 0.45	1.47 (0.64–1.77)	0.395
C20:3 n-3—(ETA) eicosatrienoic acid	0.76 ± 0.18	0.75 (0.48–1.25)	0.71 ± 0.18	0.73 (0.36–1.0)	0.82 ± 0.15	0.81 (0.66–1.15)	0.93 ± 0.18	0.90 (0.71–1.23)	0.048 *
C20:5 n-3—(EPA) eicosapentaenoic acid	0.10 ± 0.15	0.04 (0.013–0.63)	0.11 ± 0.15	0.05 (0.025–0.644)	0.11 ± 0.10	0.07 (0.021–0.385)	0.07 ± 0.07	0.05 (0.016–0.243)	0.336
C22:6 n-3—(DHA) docosahexaenoic acid	0.58 ± 0.44	0.44 (0.15–1.90)	0.79 ± 0.37	0.82 (0.34–1.42)	0.788 ± 0.40	0.79 (0.16–1.59)	0.43 ± 0.29	0.31 (0.23–1.09)	0.036 *
n-6	15.35 ± 3.02	15.12 (9.39–22.35)	14.23 ± 2.54	14.19 (10.58–20.30)	14.75 ± 1.81	14.61 (12.27–18.34)	13.82 ± 1.36	14.06 (11.60–15.54)	0.385
C18:2 n-6c—(LA) linoleic acid	14.33 ± 2.88	13.86 (8.7–20.9)	13.24 ± 2.42	13.34 (9.8–19.2)	13.64 ± 1.74	13.56 (11.4–17.0)	12.69 ± 1.34	12.95 (10.6–14.3)	0.374
C18:3 n-6—(GLA) γ-linolenic acid	0.04 ± 0.03	0.03 (0.000–0.1051)	0.04 ± 0.03	0.03 (0.0112–0.1338)	0.05 ± 0.03	0.05 (0.0083–0.1012)	0.06 ± 0.02	0.06 (0.0330–0.0920)	0.033 *
C20:2—(all Z)-eicosadienoic	0.31 ± 0.10	0.28 (0.11–0.55)	0.26 ± 0.09	0.26 (0.03–0.4)	0.34 ± 0.06	0.34 (0.20–0.46)	0.27 ± 0.02	0.27 (0.23–0.30)	0.035 *
C20:3 n-6—dihomo-γ-linoleic acid	0.04 ± 0.03	0.03 (0.01–0.11)	0.03 ± 0.02	0.03 (0.00–0.1)	0.06 ± 0.03	0.05 (0.02–0.12)	0.03 ± 0.01	0.03 (0.02–0.05)	0.129
C20:4 n-6—(ARA) arachidonic acid	0.64 ± 0.17	0.67 (0.253–0.970)	0.66 ± 0.15	0.67 (0.427–0.955)	0.67 ± 0.13	0.69 (0.439–0.830)	0.77 ± 0.12	0.74 (0.609–0.971)	0.246
PUFA	17.99 ± 3.49	17.56 (1.46–22.35)	17.24 ± 2.66	17.24 (1.64–20.30)	18.10 ± 2.36	18.16 (1.89–18.34)	16.61 ± 1.85	16.48 (1.62–15.54)	0.601
Other									
Conjugated linoleic acid (CLA) (Z,E)-9,11-octadecadienoic	0.13 ± 0.13	0.08 (0.019–0.475)	0.21 ± 0.17	0.15 (0.032–0.569)	0.18 ± 0.15	0.15 (0.027–0.589)	0.14 ± 0.08	0.12 (0.060–0.306)	0.234
Proportions									
PUFA/SFA	0.46		0.44		0.49		0.42		0.644
PUFA/MUFA	0.44		0.41		0.41		0.39		0.410
MUFA/SFA	1.00		0.96		0.88		0.93		0.407
n-6/n-3	6.27		5.02		4.76		5.25		0.034 *

SD—standard deviation, Min—minimum, Max—maximum, Me—median, *—significant differences (*p* < 0.05).

**Table 7:** Fatty acid content of breast milk (g/100 g of total FA) depending on BMI.

Fatty Acids	BMI (<18.5) n = 1		BMI (18.6–25.0) n = 32		BMI (25.1–29.9) n = 19		BMI (>30.0) n = 8		*p*
	Mean	Me	Mean ± SD	Me	Mean ± SD	Me	Mean ± SD	Me	
SFA	40.78	40.78	40.41 ± 5.64	40.53 (27.97–53.56)	38.77 ± 3.87	38.20 (32.90–47.81)	39.58 ± 5.63	39.94 (27.11–46.17)	0.462
C8:0—caprylic acid	0.13	0.13	0.17 ± 0.08	0.15 (0.065–0.333)	0.15 ± 0.06	0.15 (0.008–0.293)	0.16 ± 0.05	0.16 (0.066–0.213)	0.836
C10:0—capric acid	1.63	1.63	1.82 ± 0.51	1.74 (0.74–2.96)	1.89 ± 0.38	1.94 (1.03–2.63)	1.75 ± 0.59	1.47 (1.06–2.67)	0.585
C12:0—lauric acid	4.86	4.86	5.63 ± 1.44	5.65 (2.90–7.83)	5.44 ± 1.42	5.65 (2.28–8.92)	5.50 ± 1.64	4.89 (3.38–7.80)	0.845
C14:0—myristic acid	6.38	6.38	6.30 ± 1.35	6.51 (3.74–8.87)	5.94 ± 1.12	5.87 (3.71–8.53)	6.35 ± 1.63	6.25 (3.91–9.12)	0.762
C15:0—pentadecanoic acid	0.72	0.72	0.43 ± 0.22	0.39 (0.08–1.0)	0.40 ± 0.16	0.36 (0.15–0.69)	0.46 ± 0.19	0.43 (0.27–0.73)	0.474
C16:0—palmitic acid	18.52	18.52	17.97 ± 2.94	17.68 (12.78–27.61)	17.22 ± 1.39	16.94 (15.33–19.74)	17.46 ± 2.31	18.24 (12.06–19.33)	0.643
C17:0—margaric acid	1.07	1.07	0.85 ± 0.21	0.89 (0.40–1.36)	0.82 ± 0.18	0.79 (0.41–1.12)	0.83 ± 0.16	0.79 (0.62–1.03)	0.508
C18:0—stearic acid	6.97	6.97	6.74 ± 1.16	6.73 (4.78–9.40)	6.38 ± 0.72	6.33 (5.46–8.27)	6.48 ± 1.15	6.68 (4.03–7.62)	0.571
C20:0—arachidic acid	0.14	0.14	0.12 ± 0.07	0.10 (0.03–0.28)	0.14 ± 0.06	0.15 (0.01–0.26)	0.17 ± 0.10	0.15 (0.09–0.40)	0.206
C24:0—lignoceric acid	0.37	0.37	0.38 ± 0.08	0.38 (0.21–0.64)	0.39 ± 0.12	0.41 (0.02–0.64)	0.41 ± 0.07	0.41 (0.30–0.56)	0.739
MUFA	42.16	42.16	41.48 ± 4.50	41.16 (33.04–53.02)	43.69 ± 2.99	43.35 (37.99–48.32)	43.08 ± 3.45	42.16 (39.28–48.93)	0.259
C14:1—myristoleic acid	0.50	0.50	0.29 ± 0.18	0.24 (0.04–0.74)	0.31 ± 0.17	0.30 (0.06–0.6)	0.37 ± 0.17	0.31 (0.18–0.63)	0.380
C16:1—palmitoleic acid	3.16	3.16	2.75 ± 0.68	2.65 (1.47–4.42)	2.89 ± 0.54	2.75 (1.88–4.11)	2.88 ± 0.37	3.01 (2.19–3.33)	0.548
C18:1 n-9c—oleic acid	37.42	37.42	37.22 ± 4.50	36.25 (30.5–49.7)	39.20 ± 2.96	39.35 (32.4–44.2)	38.68 ± 3.56	38.01 (34.5–44.9)	0.225
C18:1 n-9t—elaidic acid	0.22	0.22	0.22 ± 0.14	0.18 (0.06–0.81)	0.23 ± 0.10	0.20 (0.04–0.51)	0.23 ± 0.11	0.21 (0.12–0.45)	0.634
C20:1—eicosenoic acid	0.65	0.65	0.76 ± 0.27	0.72 (0.39–1.82)	0.82 ± 0.20	0.81 (0.55–1.45)	0.72 ± 0.11	0.69 (0.61–0.91)	0.283
C22:1 n-9—erucic acid	0.22	0.22	0.25 ± 0.08	0.23 (0.16–0.51)	0.24 ± 0.09	0.23 (0.07–0.47)	0.21 ± 0.02	0.21 (0.19–0.23)	0.384
n-3	3.08	3.08	2.68 ± 0.79	2.64 (1.46–4.18)	3.20 ± 0.80	3.27 (1.89–4.74)	3.15 ± 1.20	2.97 (1.62–5.43)	0.228
C18:3 n-3—(ALA) α- linolenic acid	1.39	1.39	1.25 ± 0.64	1.05 (0.35–3.17)	1.49 ± 0.63	1.64 (0.49–2.61)	1.58 ± 0.93	1.47 (0.64–3.65)	0.488
C20:3 n-3—(ETA) eicosatrienoic acid	0.66	0.66	0.72 ± 0.17	0.72 (0.48–1.25)	0.84 ± 0.18	0.85 (0.36–1.15)	0.90 ± 0.21	0.84 (0.66–1.23)	0.011 *
C20:5 n-3—(EPA) eicosapentaenoic acid	0.16	0.16	0.09 ± 0.13	0.05 (0.013–0.630)	0.13 ± 0.16	0.06 (0.021–0.644)	0.09 ± 0.07	0.06 (0.016–0.243)	0.170
C22:6 n-3—(DHA) docosahexaenoic acid	0.87	0.87	0.62 ± 0.41	0.52 (0.15–1.90)	0.74 ± 0.44	0.63 (0.16–1.59)	0.58 ± 0.35	0.50 (0.23–1.09)	0.616
n-6	13.70	13.70	15.28 ± 3.01	15.00 (9.39–22.35)	14.18 ± 1.32	14.41 (10.58–16.19)	13.97 ± 2.15	13.34 (11.60–18.34)	0.343
C18:2 n-6c—(LA) linoleic acid	12.62	12.62	14.27 ± 2.87	13.77 (8.70–20.9)	13.09 ± 1.23	13.34 (9.8–14.8)	12.83 ± 2.07	12.09 (10.6–17.0)	0.268
C18:3 n-6—(GLA) γ-linolenic acid	0.06	0.06	0.04 ± 0.03	0.03 (0.0000–0.1051)	0.05 ± 0.03	0.05 (0.0083–0.1338)	0.06 ± 0.02	0.06 (0.0330–0.1012)	0.021 *
C20:2—(all Z)-eicosadienoic	0.22	0.22	0.31 ± 0.10	0.28 (0.11–0.55)	0.30 ± 0.09	0.30 (0.03–0.46)	0.27 ± 0.06	0.27 (0.20–0.40)	0.299
C20:3n-6—dihomo-γ-linoleic acid	0.03	0.03	0.04 ± 0.02	0.03 (0.01–0.11)	0.04 ± 0.02	0.04 (0.00–0.10)	0.04 ± 0.03	0.03 (0.02–0.12)	0.563
C20:4 n-6—(ARA) arachidonic acid	0.77	0.77	0.63 ± 0.15	0.63 (0.253–0.970)	0.69 ± 0.16	0.74 (0.427–0.955)	0.75 ± 0.13	0.75 (0.609–0.971)	0.088
PUFA	16.77	16.77	17.96 ± 3.33	17.75 (1.46–22.35)	17.37 ± 1.82	17.71 (1.89–16.19)	17.12 ± 3.18	16.31 (1.62–18.34)	0.670
Other									
Conjugated linoleic acid (CLA) (Z,E)-9,11-octadecadienoic	0.29	0.29	0.15 ± 0.14	0.12 (0.019–0.569)	0.16 ± 0.13	0.14 (0.027–0.519)	0.21 ± 0.18	0.16 (0.060–0.598)	0.392
Proportions									
PUFA/SFA	0.41		0.46		0.45		0.45		0.719
PUFA/MUFA	0.40		0.44		0.40		0.40		0.532
MUFA/SFA	0.97		1.00		0.90		0.93		0.411
n-6/n-3	4.45		6.12		4.67		4.87		0.020 *

BMI—body mass index, SD—standard deviation, Me—median, *—significant differences (*p* < 0.05).

**Table 8:** Fatty acid content of breast milk (g/100 g of total FA) depending on health.

Fatty Acids	Healthy Women (n = 49)		With Chronic Diseases Treated before Pregnancy (n = 19)		*p*
	Mean ± SD	Me (Min–Max)	Mean ± SD	Me (Min–Max)	
Saturated fatty acids (SFA)					
C8:0—caprylic acid	0.17 ± 0.1	0.15 (0.07–0.33)	0.15 ± 0.1	0.15 (0.01–0.29)	0.463
C10:0—capric acid	1.79 ± 0.5	1.82 (0.74–2.66)	1.89 ± 0.5	1.78 (1.21–2.96)	0.741
C12:0—lauric acid	5.50 ± 1.4	5.64 (2.28–7.83)	5.60 ± 1.5	5.20 (2.95–8.92)	0.952
C14:0—myristic acid	6.26 ± 1.3	6.70 (3.71–8.87)	6.11 ± 1.3	5.87 (3.74–9.12)	0.600
C15:0—pentadecanoic acid	0.44 ± 0.2	0.42 (0.15–1.00)	0.41 ± 0.2	0.36 (0.08–0.73)	0.610
C16:0—palmitic acid	18.00 ± 2.6	17.53 (14.62–27.61)	17.21 ± 2.1	17.21 (12.06–20.57)	0.579
C17:0—margaric acid	0.88 ± 0.2	0.90 (0.48–1.36)	0.80 ± 0.2	0.79 (0.40–1.12)	0.150
C18:0—stearic acid	6.80 ± 1.1	6.64 (5.24–9.40)	6.32 ± 0.9	6.40 (4.03–7.62)	0.172
C20:0—arachidic acid	0.14 ± 0.1	0.13 (0.04–0.40)	0.12 ± 0.1	0.11 (0.01–0.26)	0.267
C24:0—lignoceric acid	0.38 ± 0.1	0.39 (0.21–0.64)	0.40 ± 0.1	0.41 (0.02–0.64)	0.225
Monounsaturated fatty acids (MUFAs)					
C14:1—myristoleic acid	0.32 ± 0.2	0.27 (0.07–0.74)	0.30 ± 0.2	0.28 (0.04–0.64)	0.799
C16:1—palmitoleic acid	2.74 ± 0.6	2.69 (1.47–4.29)	2.92 ± 0.6	2.88 (1.74–4.42)	0.368
C18:1 n-9c—oleic acid	37.71 ± 3.9	37.69 (30.51–44.17)	38.51 ± 4.0	37.39 (32.41–49.67)	0.730
C18:1 n-9t—elaidic acid	0.24 ± 0.1	0.20 (0.06–0.81)	0.19 ± 0.1	0.18 (0.04–0.45)	0.077
C20:1—eicosenoic acid	0.76 ± 0.3	0.72 (0.39–1.82)	0.78 ± 0.2	0.74 (0.56–1.45)	0.500
C22:1 n-9—erucic acid	0.24 ± 0.1	0.23 (0.16–0.51)	0.25 ± 0.1	0.23 (0.07–0.47)	0.697
n-3 polyunsaturated fatty acids (n-3 PUFAs)					
C18:3 n-3—(ALA) α-linolenic acid	1.33 ± 0.6	1.17 (0.49–2.61)	1.43 ± 0.8	1.30 (0.35–3.65)	0.845
(ETA) C20:3 n-3—eicosatrienoic acid	0.79 ± 0.2	0.78 (0.48–1.25)	0.78 ± 0.2	0.77 (0.36–1.23)	0.822
C20:5 n-3—(EPA) eicosapentaenoic acid	0.09 ± 0.1	0.05 (0.01–0.63)	0.12 ± 0.1	0.06 (0.02–0.64)	0.294
C22:6 n-3—(DHA) docosahexaenoic acid	0.60 ± 0.3	0.52 (0.15–1.32)	0.74 ± 0.5	0.61 (0.20–1.90)	0.337
n-6 polyunsaturated fatty acids (n-6 PUFAs)					
C18:2 n-6c—(LA) linoleic acid	13.60 ± 2.5	13.41 (8.67–20.12)	13.78 ± 2.3	13.56 (10.61–20.87)	0.893
C18:3 n-6—(GLA) γ-linolenic acid	0.05 ± 0.0	0.05 (0.00–0.13)	0.04 ± 0.00	0.03 (0.01–0.09)	0.219
C20:2—(all Z)-eicosadienoic	0.30 ± 0.1	0.28 (0.11–0.55)	0.31 ± 0.1	0.31 (0.03–0.46)	0.393
C20:3 n-6—dihomo-γ-linoleic acid	0.04 ± 0.0	0.03 (0.01–0.11)	0.04 ± 0.00	0.04 (0.00–0.12)	0.472
C20:4 n-6—(ARA) arachidonic acid	0.65 ± 0.2	0.68 (0.25–0.95)	0.69 ± 0.2	0.67 (0.43–0.97)	0.463
Other					
Conjugated linoleic acid (CLA) (Z,E)-9,11-octadecadienoic acid	0.18 ± 0.2	0.13 (0.03–0.60)	0.14 ± 0.1	0.12 (0.02–0.52)	0.519

SD—standard deviation, Min—minimum, Max—maximum, Me—median.

## References

[1] Roszkowska R., Taranta-Janusz K., Wasilewska A. (2014). Rola wczesnego programowania metabolicznego w patogenezie chorób cywilizacyjnych. Dev. Period Med..

[2] Lauritzen L., Carlson S.E. (2011). Maternal fatty acid status during pregnancy and lactation and relation to newborn and infant status. Matern. Child Nutr..

[3] Lauritzen L., Brambilla P., Mazzocchi A., Harsløf L., Ciappolino V., Agostoni C. (2016). DHA effects in brain development and function. Nutrients.

[4] Uauy R., Dangour A.D. (2006). Nutrition in brain development and aging: Role of essential fatty acids. Nutr. Rev..

[5] Andreas N.J., Kampmann B., Mehring Le-Doare K. (2015). Human breast milk: A review on its composition and bioactivity. Early Hum. Dev..

[6] Ballard O., Morrow A.L. (2013). Human milk composition: Nutrients and bioactive factors. Pediatr. Clin. North Am..

[7] Innis S.M. (2014). Impact of maternal diet on human milk composition and neurological development of infants. Am. J. Clin. Nutr..

[8] Innis S.M. (2013). Maternal Nutrition, Genetics, and Human Milk Lipids. Curr. Nutr. Rep..

[9] Babiszewska M., Ziomkiewicz A., Wesołowska A. (2016). Tłuszcz mleka kobiecego—Skład, synteza i funkcja kwasów tłuszczowych oraz pochodnych lipidów. Pediatr. Pol..

[10] Szlagatys-Sidorkiewicz A., Brodzicki J. (2008). Mleko Kobiece. Aktualny Stan Wiedzy.

[11] Euro WHO. https://www.who.int/europe/news-room/fact-sheets/item/a-healthy-lifestyle---who-recommendations.

[12] Gaete G.M., Atalah S.E. (2003). Niveles de LC-PUFA n-3 en la leche maternal después de incentivar el consumo de alimentosmarinos. Rev. Child Pediatr..

[13] Bortolozo E.A.F.Q., Sauer E., Santos M.S., Baggio S.R., dos Santos G., Farago P.V., Bileski Cândido L.M., Pilatti L.A. (2013). Supplementation with the omega-3 docosahexaenoic acid: Influence on the lipid composition and fatty acid profile of HM. Rev. Nutr..

[14] Nishimura R.Y., Barbieiri P., de Castro G.S., Jordão A.A., da Silva Castro Perdoná G., Sartorelli D.S. (2014). Dietary polyunsaturated fatty acid intake during late pregnancy affects fatty acid composition of mature breast milk. Nutrition.

[15] Pedersen L., Lauritzen L., Brasholt M., Buhl T., Bisgaard H. (2012). Polyunsaturated fatty acid content of mother’s milk is associated with childhood body composition. Pediatr. Res..

[16] Sánchez-Hernández S., Esteban-Muñoz A., Giménez-Martínez R., Aguilar-Cordero M.J., Miralles-Buraglia B., Olalla-Herrera M. (2019). A Comparison of Changes in the Fatty Acid Profile of Human Milk of Spanish Lactating Women during the First Month of Lactation Using Gas Chromatography-Mass Spectrometry. A Comparison with Infant Formulas. Nutrients.

[17] Giuffrida F., Fleith M., Goyer A., Samuel T.M., Elmelegy-Masserey I., Fontannaz P., Cruz-Hernandez C., Thakkar S.K., Monnard C., De Castro C.A. (2022). Human milk fatty acid composition and its association with maternal blood and adipose tissue fatty acid content in a cohort of women from Europe. Eur. J. Nutr..

[18] Samur G., Topcu A., Turan S. (2009). Trans Fatty Acids and Fatty Acid Composition of Mature Breast Milk in Turkish Women and Their Association with Maternal Diet’s. Lipids.

[19] Sherry C.L., Oliver J.S., Marriage B.J. (2015). Docosahexaenoic acid supplementation in lactating women increases breastmilk and plasma docosahexaenoic acidconcentrationsand alters infant omega 6: 3 fatty acid ratio. Prostaglandins Leukot. Essent. Fat Acids.

[20] Ogunleye A., Fakoya A.T., Niizeki S., Tojo H., Sasajima I., Kobayashi M., Tateishi S., Yamaguchi K. (1991). Fatty acid composition of breast milk from Nigerian and Japanese women. J. Nutr. Sci. Vitam..

[21] Siziba L., Chimhashu T., Siro S.S., Ngounda J.O., Jacobs A., Malan L., Smuts C.M., Baumgartner J. (2020). Breast milk and erythrocyte fatty acid composition of lactating women residing in a peri-urban South African township. Prostaglandins Leukot. Essent. Fat Acids.

[22] Sauerwald T.U., Demmelmair H., Koletzko B. (2001). Polyunsaturated Fatty Acid Supply with Human Milk. Lipids.

[23] Fu Y., Liu X., Zhou B., Jiang A.C., Chai L. (2016). An updated review of worldwide levels of docosahexaenoic and arachidonic acid in human breast milk by region. Public Health Nutr..

[24] Jimenez E.Y., Mangani C., Ashorn P., Harris W.S., Maleta K., Dewey K.G. (2015). Breast milk from women living near Lake Malawi is high in docosahexaenoic acid and arachidonic acid. Prostaglandins Leukot. Essent. Fat Acids.

[25] Brenna J.T., Varamini B., Jensen R.G., Diersen-Schade D.A., Boettcher J.A., Arterburn L.M. (2007). Docosahexaenoic and arachidonic acid concentrations in human breast milk worldwide. Am. J. Clin. Nutr..

[26] Bzikowska-Jura A., Czerwonogrodzka-Senczyna A., Jasińska-Melon E., Mojska H., Olędzka G., Wesołowska A., Szostak-Węgierek D. (2019). The Concentration of Omega-3 Fatty Acids in Human Milk Is Related to Their Habitual but Not Current Intake. Nutrients.

[27] Kim H., Kang S., Jung B.M., Yi H., Jung J.A., Chang N. (2017). Breast milk fatty acid composition and fatty acid intake of lactating mothers in South Korea. Br. J. Nutr..

[28] Wang L., Shimizu Y., Kaneko S., Hanaka S., Abe T., Shimasaki H., Hisaki H., Nakajima H. (2000). Comparison of the fatty acid composition of total lipids and phospholipids in breast milk from Japanese women. Pediatr. Int..

[29] Helland I.B., Saarem K., Saugstad O.D., Drevon A. (1998). Fatty acid composition in maternal milk and plasma during supplementation with cod liver oil. Eur. J. Clin. Nutr..

[30] Puca D., Estay P., Valenzuela C., Munoz Y. (2021). Effect of omega-3 supplementation during pregnancy and lactation on the fatty acid composition of breast milk in the first months of life: A narrative review. Nutr. Hosp..

[31] Di Villarosa do Amaral Y.N., Marano D., Lopes da Silva L.M., Guimarães A.C.L.D., Moreira M.E.L. (2017). Are There Changes in the Fatty Acid Profile of Breast Milk with Supplementation of Omega-3 Sources? A Systematic Review. Rev. Bras. Ginecol. Obs..

[32] Much D., Brunner S., Vollhardt C., Schmid D., Sedlmeier E.M., Brüder M., Heimberg E., Bartke N., Boehm G., Bader B.L. (2013). Breast milk fatty acid profile in relation to infant growth and body composition: Results from the INFAT study. Pediatr. Res..

[33] Mäkelä J., Linderborg K., Niinikoski H., Yang B., Lagström H. (2013). Breast milk fatty acid composition differs between overweight and normal weight women: The STEPS Study. Eur. J. Nutr..

[34] Warstedt K., Furuhjelm C., Fälth-Magnusson K., Fagerås M., Duchén K. (2016). High levels of omega-3 fatty acids in milk from omega-3 fatty acid-supplemented mothers are related to less immunoglobulin E-associated disease in infancy. Acta Paediatr..

[35] Xiang M., Harbige L.S., Zetterstrom R. (2005). Long-chain polyunsaturated fatty acids in Chinese and Swedish mothers: Diet, breast milk and infant growth. Acta Paediatr..

[36] Storck Lindholm E., Strandvik B., Altman D., Möller A., Palme Kilander C. (2013). Different fatty acid pattern in breast milk of obese compared to normal-weight mothers. Prostaglandins Leukot. Essent. Fat Acids.

[37] Miliku K., Duan Q.L., Moraes T.J., Becker A.B., Mandhane P.J., Turvey S.E., Lefebvre D.L., Sears M.R., Subbarao P., Field C.J. (2019). Human milk fatty acid composition is associated with dietary, genetic, sociodemographic, and environmental factors in the CHILD Cohort Study. Am. J. Clin. Nutr..

[38] Marın M.C., Sanjurjo A., Rodrigo M.A., de Alaniz M.J.T. (2005). Long-chain polyunsaturated fatty acids in breast milk in La Plata, Argentina: Relationship with maternal nutritional status. Prostaglandins Leukot. Essent. Fat Acids.

[39] Nayak U., Kanungo S., Zhang D., Colgate E.R., Carmolli M.P., Dey A., Alam M., Manna B., Nandy R.K., Kim D.R. (2017). Influence of maternal and socioeconomic factors on breast milk fatty acid composition in urban, low-income families. Matern. Child Nutr..

[40] Wang Y., Jones P.J.H. (2004). Dietary conjugated linoleic acid and body composition. Am. J. Clin. Nutr..

[41] Nishimura R.Y., de Castro G.S.F., Jordăo J.J., Sartorelli D.S. (2013). Breast milk fatty acid composition of women living far from the coastal area in Brazil. J. Pediatr..

[42] Precht D., Molkentin J. (1997). Effect of feeding on conjugated cis delta 9, trans delta 11-octadecadienoic acid and other isomers of linoleic acid in bovine milk fats. Nahrung.

[43] Martysiak-Żurowska D., Kiełbratowska B., Szlagatys-Sidorkiewicz A. (2018). The content of conjugated linoleic acid and vaccenic acid in the breast milk of women from Gdansk and the surrounding district, as well as in, infant formulas and follow up formulas. Nutritional recommendation for nursing women. Dev. Period Med..

